# Pathways and obstacles to social recovery following the elimination of SARS-CoV-2 from Aotearoa New Zealand: a qualitative cross-sectional study

**DOI:** 10.1093/pubmed/fdab394

**Published:** 2022-01-07

**Authors:** Nicholas J Long, Nayantara Sheoran Appleton, Sharyn Graham Davies, Antje Deckert, Edmond Fehoko, Eleanor Holroyd, Nelly Martin-Anatias, Rogena Sterling, Susanna Trnka, Laumua Tunufa’i

**Affiliations:** Department of Anthropology, London School of Economics and Political Science, London WC2A 2AE, UK; Centre for Science in Society, Victoria University of Wellington, Wellington 6012, New Zealand; School of Languages, Literatures, Cultures, and Linguistics, Monash University, Clayton 3800, Australia; School of Social Sciences and Public Policy, Auckland University of Technology, Auckland 1010, New Zealand; School of Social Sciences and Public Policy, Auckland University of Technology, Auckland 1010, New Zealand; School of Māori Studies and Pacific Studies, University of Auckland, Auckland 1010, New Zealand; Faculty of Health and Environmental Sciences, Auckland University of Technology, Auckland 0627, New Zealand; School of Social Sciences and Public Policy, Auckland University of Technology, Auckland 1010, New Zealand; University of Waikato, Hamilton 3240, New Zealand; School of Social Sciences, University of Auckland, Auckland 1010, New Zealand; School of Social Sciences and Public Policy, Auckland University of Technology, Auckland 1010, New Zealand

**Keywords:** COVID-19, health policy, pandemic, sense of normality, social isolation, social relationships

## Abstract

**Background:**

Many public health experts have claimed that elimination strategies of pandemic response allow ‘normal social life’ to resume. Recognizing that social connections and feelings of normality are important for public health, this study examines whether, and for whom, that goal is realized, and identifies obstacles that may inhibit its achievement.

**Methods:**

Thematic analysis of narratives obtained via a qualitative cross-sectional survey of a community cohort in Aotearoa | New Zealand.

**Results:**

A majority of participants reported that life after elimination was ‘more or less the same’ as before the pandemic. Some became more social. Nevertheless, a sizeable minority reported being less social, even many months after elimination. Key obstacles to social recovery included fears that the virus was circulating undetected and the enduring impact of lockdowns upon social relationships, personal habits and mental health. Within our sample, old age and underlying health conditions were both associated with a propensity to become less social.

**Conclusions:**

Elimination strategies can successfully allow ‘normal social life’ to resume. However, this outcome is not guaranteed. People may encounter difficulties with re-establishing social connections in Zero-COVID settings. Measures designed to overcome such obstacles should be an integral part of elimination strategies.

## Introduction

Since the onset of the COVID-19 pandemic, the respective merits of mitigation and elimination strategies have been hotly debated in public health circles.^1^ In mitigation, the virus continues to circulate, albeit at reduced levels, due to non-pharmaceutical interventions (NPIs). Elimination, or ‘Zero-COVID’, involves initial deployment of stringent NPIs with a view to reducing community spread to zero; further outbreaks are then guarded against via strict border controls. Though vaccinations are playing an increasingly important role in international responses to COVID-19, the mitigation-elimination debate remains salient—both when vaccination rates remain low,^2^ and when planning for future pandemics.^3^

Notwithstanding technical questions regarding elimination’s feasibility (which may prove difficult in developing countries or when faced with highly transmissible SARS-CoV-2 variants), proponents of each strategy are motivated by competing understandings of how best to protect the public good. Advocates of mitigation argue that the economic damage wrought by the stringent lockdowns and border closures necessary to secure Zero-COVID status would have long-term repercussions for public health.^1^ Supporters of elimination counter that it minimizes COVID-19 fatalities, supports economic recovery and involves less overall restriction of civil liberties.^1^^,^^4^ Elimination is also seen as having social and psychological benefits, often couched in the idiom of allowing ‘normal social life’ to resume’.^5^^,^^6^ These benefits are themselves a public health matter, since a sense of ‘normality’ and a high quantity and quality of social relations are associated with improved physical and mental health.^7^^,^^8^

Current evidence supports both the clinical and economic arguments in favour of elimination.^4^^,^^8^^,^^9^ However, although research indicates that people living through *mitigation* strategies have fewer social contacts once ‘lockdown’ restrictions are lifted than they did pre-pandemic,^10^ little is yet known about whether a ‘normal’ or satisfying social life returns after elimination. Measuring such attributes requires a qualitative, social constructionist approach, since feelings of ‘normality’ and ‘satisfaction’ principally inhere in subjective evaluations of a practice or situation compared to an imagined baseline of ‘normal activity’; these evaluations are then expressed through narrative in a process of ‘narrative sense-making’.^11^ Generated by individuals whose yardsticks of evaluation are both constantly evolving and distinct from those of others (even as they are to some degree co-constructed), each narrative must be engaged with qualitatively, and on its own terms, whilst also remaining attentive to patterns across different cases. In this paper, we thus develop a thematic analysis of narratives describing life in a Zero-COVID setting—New Zealand between February and August 2021—to add greater nuance to existing claims regarding the extent and ease of social recovery following viral elimination.

## Methods

### Site selection

New Zealand has received international acclaim for the success of its elimination strategy. Elimination first occurred in May 2020, following a 49-day national lockdown.^12^ On 8 June 2020, the country moved to ‘Alert Level 1’: activities could resume without restriction—although enhanced record-keeping was recommended. Small community outbreaks in August 2020 and February 2021 led to short periods of enhanced restriction, mostly in Auckland. Nevertheless, until the Delta variant triggered a further nationwide lockdown in August 2021, New Zealand had enjoyed many months of freedom from COVID-19 restrictions. With vaccine rollout progressing slowly during this time (only 18% of the population were double vaccinated by the time of this study),^13^ elimination was the cornerstone on which prospects of social recovery depended. Indeed, the New Zealand government cited ‘get[ting] back to a sense of normality’ as a reason to persist with its elimination strategy.^14^ New Zealand is thus an instructive site in which to investigate how post-elimination social life has been experienced.

### Research design

Following Cole and Knowles’ argument that every ‘exploration of an individual life-in-context brings us that much closer to understanding the complexities of lives in communities’,^15^ this study sought to document the *range* of possible experiences that people could have following elimination, using thematic analysis of respondents’ narratives to identify key dynamics underpinning different behavioural pathways.

Recognizing the flexibility, scalability and richness of online surveys as a method of gathering qualitative data,^16^ we advertised a self-administered online survey via nationwide Facebook and Instagram campaigns between 18 August and 25 August 2021, with bespoke campaigns targeted at men and younger age groups to heighten variation within the sample. These campaigns recruited 225 participants. The study was also advertised to a database of 1417 contacts who had participated in previous surveys, themselves recruited via campaigns intended to maximize variation. 815 of these contacts completed the survey (a response rate of 57.5%).

The final respondent pool contained a wide breadth of ages and regions of residence but, despite attempts to maximize variation, contained disproportionate numbers of women, New Zealand European/Pākehā people and university graduates—as is often the case with survey research in New Zealand.^17^

Respondents were asked to comment on multiple aspects of New Zealand’s pandemic response, to evaluate how much their lives had changed since before the pandemic, and to provide narrative elaborations (see Supplemental Material, Annex 1).

### Analysis

Following an unstructured familiarization phase, respondents’ answers to open-ended questions were independently coded by two researchers, using both anticipated themes and emergent themes that were discovered in the data and refined via discussion. Data were checked against an initial framework and refinements made as necessary. We also conducted a descriptive statistical analysis to examine whether certain codes were associated with respondent characteristics such as gender, age, ethnicity, education status, household size or medical vulnerability to COVID-19 (see Supplemental Material, Annex 2).

## Results

966 of the 1040 survey respondents (92.9%) answered Q6.4—a ‘tick-box’ question about how their social life compared to life before the pandemic. 546 respondents (52.5% of the total) provided narrative elaborations. Thematic analysis of these narratives revealed three overarching patterns of social behaviour in the wake of elimination.

### Returning to a pre-pandemic ‘normal’

531 respondents indicated their friendships and social life had been ‘more or less the same’ over the previous six months (i.e. from February to August 2021) as before the pandemic. When elaborating on their answers, most attested that nothing had changed (Table 1 – Quote 1). They described level 1 as allowing a return to normality (Quote 2), and feeling grateful and ‘lucky’ that the New Zealand government had adopted an elimination strategy (Quotes 3 and 4). Some suggested the social gains of life at levels 1 and 2 justified the ‘sacrifice’ of lockdowns (Quote 5). Several mentioned that elimination had allowed them to feel ‘safe’ (Quotes 3 and 6), alleviating their feelings of ‘fear’ (Quote 7). One respondent, who had spent three months in the UK, which has adopted a mitigation strategy, contrasted the ‘normality’ of Zero-COVID New Zealand with the ‘frightening’ feeling of life in Britain (Quote 8). Only occasionally was the ‘normality’ of social life linked to a wilful blindness towards the pandemic (Quote 9). Interestingly, although most respondents were supportive of the vaccination programme elsewhere in the survey, none mentioned it contributing to their experiences of social recovery.

**Table 1:** Returning to pre-pandemic normality

Quote 1	My friendships and social life were the same before and after lockdown. My social circle is small anyway so it is not difficult to maintain.	Māori woman, 40s
Quote 2	Things returned to normal once we returned to level 1.	Pākehā woman, 30s
Quote 3	Thanks to the government keeping us safe, we’ve been able to enjoy most of what we usually do.	Pākehā woman, 60s
Quote 4	We are lucky to have an elimination strategy, so apart from the initial lockdown itself very little had to change.	Pākehā woman, 30s
Quote 5	Every time we are at level 1 or 2 then all of our social life explodes again which is lovely – I’m willing to sacrifice here and there in a level 3 or 4 in order for the huge gains we get the rest of the time. We’ve been able to take holidays around NZ, go to arts and sports events, I’ve been able to sing in my symphonic choir with audiences of nearly 3000, go to big birthday events, rugby, parties, and big work events. It’s an incredibly fortunate life so far.	Pākehā woman, 50s
Quote 6	Things appeared to be normal and we felt safe	Pākehā man, 60s
Quote 7	Having the freedom of choice to again visit friends and family without any fear and go have a normal social life.	Pākehā woman, 70s
Quote 8	In NZ everything was normal till this new lockdown so normal life prevailed. In the UK everything was different – unable to see my friends until the UK lockdown relaxed. Even then I was frightened of putting folk in danger. Kept wearing my mask	Pākehā woman, 60s
Quote 9	By turning off all media life can carry on without all the lies and one sided rubbish	Māori and Pākehā man, 50s
Quote 10	I am more mindful of sickness/illness and how my family may impact the health of others and vice versa. I am much more conscious about my movements and that of my family.	Māori and Pākehā woman, 30s
Quote 11	More or less the same but cognizant of public health precautions – washing hands, etc.	Pākehā man, 60s
Quote 12	We don’t have a raging social life with a young child and a baby on the way. But we’ve been happy to go to theatres or cinemas. I don’t have any friends who are border workers and don’t get out too much in level 2 anyway.	European woman, 40s
Quote 13	Although some of us have different opinions about the govt. and their decisions re pandemic, our friendships are strong and we can have robust discussions without getting personal.	Pākehā woman, 60s

Eleven respondents, some of whom indicated that their social life had become ‘a little different’, described minor changes resulting from heightened awareness of health and hygiene as opposed to substantive shifts in social activity (Quotes 10 and 11). Nevertheless, several reports of a ‘return to normality’ were haunted by a sense of contingency, with respondents indicating that life might have been less normal if they had friends who were border workers (Quote 12), or if they were less adept at handling differences of opinion within their relationships (Quote 13). This indicates a recognition that the post-elimination context may put strain on certain relationships.

### Becoming more social

95 respondents described intensified social activities following elimination. Some framed this as a response to lockdown, couched in the idiom of ‘making up for lost time’ (Table 2 – Quote 1). Others explained that the pandemic had revealed the fragility of social freedoms (Quote 2), and, indeed, human life (Quote 3), inspiring them to prioritize friendships and social activities more than previously (Quotes 4 and 5), and to appreciate their loved ones more (Quote 6).

**Table 2:** Becoming more social

Quote 1	I have been going out as much as possible when not in lockdown to make up for lost time	Asian and Pākehā woman, 20s
Quote 2	I think we have embraced our friends and social life with much more gusto, cos you just don’t know when we’ll be locked down again. We’ve been doing quite a lot more domestic travelling and local activities. Also trying to support local businesses.	Australian woman, 20s
Quote 3	More social events as don’t want to miss out because people could die anytime.	Pākehā woman, 20s
Quote 4	I have made more effort to join in	Pākehā man, 60s
Quote 5	I’ve built stronger friendships with my flatmates and been far more willing to go out and do things. I think this has partially been in an effort to make the most of the time we are not in lockdown. Prior to the pandemic I often turned down social events to study.	Pākehā woman, 20s
Quote 6	More appreciative of friends who can keep company through hard things like lockdown. Some older friendships refreshed because of time and drive to reconnect – rather than ‘leaving it to another day’.	Pākehā woman, 50s
Quote 7	Our neighbourhood formed closer friendships during previous lockdown (socially distanced afternoon teas on the street), and we have continued supportive closer relationships.	Pākehā woman, 60s
Quote 8	Got closer to most people. Am more honest about my emotions	Pākehā woman, 50s
Quote 9	I think my social life has been better with more meaningful relationships	Pākehā woman, 30s

In several cases, being ‘locked down’ had afforded opportunities for new friendships to arise in the local community, and these had persisted into the post-elimination period (Quote 7). Others indicated the pandemic had led them to forge closer relationships, allowing them to be more honest about their emotions (Quote 8), or rendering friendships more ‘meaningful’ (Quote 9).

### Becoming less social

253 respondents presented narratives in which life after elimination was associated with a decline in the quantity or quality of their social relationships. In 24 cases, this was explicitly linked to the border closures integral to the elimination strategy (Table 3 – Quote 1). For the majority, however, their social lives *within New Zealand* had changed. They reported changes in activities (‘socializing less’ or spending less time in public places – Quotes 2 and 3), changes in character (becoming ‘less social’ – Quote 4), and an overall sense of their world ‘having shrunk’ (Quote 5).

**Table 3:** Becoming less social

Quote 1	Can’t visit my best friend in Australia who has gone through a really tough time. We’ve drifted apart a bit because of this.	Māori and Pākehā woman, 30s
Quote 2	I seem to go out less to meet up with friends.	European woman, 50s
Quote 3	It has been harder to reconnect with others. My attendance/participation in usual activities (e.g. going to church) has changed, I don’t feel so well connected. I’m wary of larger gatherings, not going to movies etc. as much as before.	Pākehā man, 50s
Quote 4	I’m less social now and have to push myself to work in friendships and relationships outside my bubble	Pākehā woman, 50s
Quote 5	I am spending more quality time with fewer people. I’m mindful of my world having shrunk quite considerably since COVID.	European woman, 40s
Quote 6	My husband has become reluctant to socialize around strangers. He doesn’t trust them to keep him safe. Therefore we do not go out very much. Friends are more careful about visiting if they have a cold etc.	Pākehā woman, 70s
Quote 7	I keep away from large groups as I would hate to spread covid to my tiny community.	Pacific and Pākehā woman, 40s
Quote 8	I’m way less social than I used to be. I feel anxious and stressed all the time, and then staying at home leaves me depressed, which makes me less likely to want to socialize. I haven’t seen many friends at all this year.	Pākehā man, 30s
Quote 9	It’s been very hard to maintain social connections because events keep getting cancelled. I live by myself, and I now spend a lot of time by myself...	Pākehā woman, 30s
Quote 10	Minimal due to lockdown fatigue, i.e. the effect of lockdown have drained all my energy and I don’t have the energy to socialize. Also planning is very difficult.	Pākehā woman, 50s
Quote 11	I go out less socially – not from fear of COVID just from habit. More time spent at home since last lockdown.	Pākehā non-binary person, 30s
Quote 12	I’ve lost 2 friends of more than 20 years standing. We no longer speak as we reacted to lockdown in very different ways. One was a rulebreaker and I could not cope with this lack of morality. The other became very stressed and turned very nasty, so I had to back off and have not been keen to go back.	Pākehā woman, 50s
Quote 13	A lot of people kind of withdrew into their family life during lockdown and not all of them have been as social afterwards.	Māori and Pākehā man, 30s
Quote 14	I have agreed to end a friendship of long standing – partly due to a growing divide in how we see government, societal, media and our individual responses to the pandemic. The friend I am thinking of has tried to share conspiracy theories with me, and I have tried to stay alongside and understand why she is attracted to that thinking, but it proved too much for us both.	Māori and Pākehā woman, 50s
Quote 15	I see people less often than before, even talk less often as friends and family my age stay home more and have less to talk about.	Pākehā woman, 60s
Quote 16	I have tended to focus on fewer more important relationships, and not so much the ‘lite’ friendships.	Pākehā woman, 30s
Quote 17	A few friends fell away & several more grew closer. Socializing has been smaller, more often one-on-one & tend to talk more deeply	Pākehā woman, 30s
Quote 18	I indulge my introvert side more. I no longer feel I ‘should’ make an effort to, e.g., go out into the city or attend events if I don’t really feel like it. It’s fine to stay home and stay safe, and use less petrol etc. too.	European woman, 60s
Quote 19	As I have ME/CFS I am already socially isolated and the pandemic has exacerbated this and I feel like nobody cares about me.	Pākehā woman, 40s
Quote 20	Definitely feel flatter, poorer mental health, just less to look forward to and a sense that anything I plan could be cancelled, plus friendships more fragmented	Pākehā woman, 20s

For some respondents, these changes were linked to fear that SARS-CoV-2 might have entered New Zealand and be in circulation, despite announcements that community transmission had been eliminated. They worried that, by socializing, they might either contract COVID-19 (Quote 6) or pass it to others (Quote 7). Others described how the anxiety and stress they had experienced during the pandemic had triggered feelings of depression that then impeded them from undertaking social activities (Quote 8). Such anxieties could have knock-on consequences for others, with a diminished social life sometimes arising from frequent cancellations (Quote 9).

In other cases, the change in social patterns was presented as a consequence of the 7-week lockdown in March–May 2020 (and, in some cases, subsequent local lockdowns). One respondent reported ‘lockdown fatigue’ (Quote 10), whereas others explained lockdown had ‘habituated’ them to staying at home (Quote 11). Some friendships had been strained during lockdown due to disagreements over rule-breaking (Quote 12), whereas other respondents felt that the lockdown had led people to ‘withdraw into family life’ at the expense of other relationships (Quote 13). Comparable dynamics were sometimes reported as having arisen even after lockdown: disagreements over vaccine uptake or the government’s COVID response had caused some people to sever ties with friends (Quote 14), whereas those whose friends had a propensity to ‘stay at home’—for whatever reason—noted that those relationships now felt thinner, with less to talk about (Quote 15).

Not all respondents viewed such changes as negative. Some appreciated being able to focus on their ‘most important’ relationships (Quotes 3 and 16), the ‘deeper’ conversation afforded by smaller gatherings (Quote 17), or being able to ‘indulge their introvert side’ (Quote 18). For others, however, the loss of connection had fostered feelings of isolation (Quote 19) and deteriorating mental health (Quote 20).

### Response distribution

The three patterns described above could be observed amongst respondents of all backgrounds. Descriptive statistical analysis (Supplemental Material, Annex 2) did not indicate any significant associations with ethnicity, education status or residence size. There were, however, notable associations with health status and age. Respondents with underlying conditions were more likely to have become less social and less likely to have become more social than those without such conditions. Similarly, those in younger age brackets were more likely to have become more social, and those in older age brackets more likely to have become less social—although this pattern may partly reflect the increased prevalence of underlying health conditions in older age groups. There was also a strong association with gender: women were more likely to report having become more social *or* having become less social, and men more likely to report continuity. This finding may reflect longstanding gender roles in Western societies, in which women have often been deemed responsible for thinking about and managing social relationships and thus potentially more inclined to detect and report changes in their social networks.^18^

## Discussion

### Main findings of this study

Our findings indicate that elimination strategies can indeed allow many people to regain a sense of ‘normality’ within their social lives, even inspiring them to cultivate social relationships more actively than ever before. Such positive outcomes, however, are not guaranteed: many respondents reported lower levels of social contact after the virus had been eliminated than before the pandemic. Moreover, becoming less social was associated with older age and underlying health conditions, suggesting that those most vulnerable to COVID-19 may be least able to achieve social recovery.

As our thematic analysis reveals, the shrinkage of a social network is not always undesirable: it need not equate to ‘loneliness’, and may even be experienced as a relief. Nevertheless, research in New Zealand and beyond points to strong correlations between the number and quality of social relationships and overall physical and mental health.^19–22^ There are also known psychological benefits associated with living in a world that feels ‘normal’.^7^ Enabling people to restore or expand their pre-pandemic social networks is thus a public health imperative.

Elimination strategies can be improved by anticipating and mitigating against common obstacles to social recovery that arise in post-elimination settings. Our study identified two. First is the ongoing fear of contagion, which is not necessarily eliminated with the virus—especially when a pandemic continues to rage internationally. In addressing such fear, policy makers must strike a delicate balance between promoting appropriate levels of caution (e.g. using contact tracing apps) and encouraging people to take advantage of hard-won freedoms. Public health messaging should champion adjustments made to make public venues COVID-secure (e.g. by increasing airflow), promote low-risk forms of social contact (e.g. meeting outdoors) and emphasize that reconnecting with others is itself a public health good, perhaps harnessing the tropes of ‘kindness’ and ‘togetherness’ that underpinned New Zealand’s initial messaging around lockdown.^23^

Second, the case of New Zealand shows how even relatively short lockdowns, when stringent enough to achieve elimination, can have long-term impacts on relationships, personal habits and mental health—and, by extension, social networks. Funding of mental health services (including systemic psychotherapies) should thus be increased; indeed, in New Zealand this has been a priority since well before the pandemic.^24^ Healthcare providers could encourage volunteering and other forms of social prescribing.^25^^,^^26^ Public health messaging should highlight the value of repairing interpersonal tensions that have arisen during the pandemic, disseminate advice on how to do this, and openly acknowledge that ‘returning to normal’ may need to be undertaken consciously and effortfully, rather than occurring automatically.

### What is already known on this topic

Public health scholars, politicians and journalists advocating for elimination frequently claim it enables a ‘return to normality’ and facilitates social recovery, sometimes citing New Zealand as an example.^5^^,^^14^^,^^27^^,^^28^ The evidence underpinning such claims is either anecdotal or based on thin, quantitative, measures of activity that overlook how post-elimination life is subjectively experienced.^29^^,^^30^ Few studies have addressed this latter concern. Research immediately following New Zealand’s 2020 lockdown documented high levels of aversion to mixing with strangers,^31^ uncertainty about the trajectory of the pandemic,^32^ and worsening mental health.^33–35^ However, little is known about how long such dynamics persist.

### What this study adds

To our knowledge, this is the first study to examine how life in any elimination setting has been experienced more than 6 months after elimination, let alone to capture those experiences in people’s own words. Although our study generally supports arguments in favour of elimination strategies, it adds nuance by identifying several obstacles that may impede social recovery. Foremost amongst these are ongoing anxieties that SARS-CoV-2 might be circulating undetected, and the long-term repercussions that lockdowns have had on people’s mental health, inclinations to be sociable, and friendships. Additional obstacles include stigma against border workers, disagreements over the pandemic response, and hopelessness linked to cancelled plans and uncertain futures.

Future research should investigate whether comparable patterns are observed in settings that have achieved elimination in different ways—such as Taiwan, which prioritized contact tracing over lockdowns.^36^ Such research would usefully inform decisions over what *kind* of elimination strategy governments should aspire to. Future work should also examine perspectives missing from this study, the design of which precluded access to under-18s, those too economically disadvantaged to have internet access, and non-English speakers. In New Zealand, more research is needed on Māori and Pacific experiences, given the relatively small numbers participating in the survey, the long-standing health disparities and structural inequities affecting these groups, and their disproportionate vulnerability to COVID-19.^37^^,^^38^

### Limitations of this study

Our survey’s cross-sectional nature meant the narrative sense-making we recorded was occurring at a specific moment in time. By happenstance, community outbreaks of the Delta variant led to a new lockdown just before our survey launched. Pessimism may have led some respondents to exaggerate what had been lost since before the pandemic and others to romanticize life at level 1.

Although thematic analysis allowed us to identify three post-elimination behavioural pathways and the causal logics underpinning them, we cannot be certain of their relative prevalence amongst New Zealand’s public. Given the high proportion of respondents identifying as women (76.6%) and reporting underlying health conditions (31.7%), and a mean age (48.8) above the national average (37.2), the pathway of ‘becoming less social’ is probably overrepresented in our results. Nevertheless, although a statistically representative study would better delineate the scale of the challenges, the high volume of such narratives enabled us to identify a broad range of dynamics that can obstruct social recovery, and to suggest specific measures and messaging that could have better supported the public in their transition to post-elimination life.

## Conclusion

Where possible, governments should consider elimination strategies of pandemic response on the grounds that they can enable social recovery, as well as guarding against excess mortality and limiting economic damage. Nevertheless, pandemic planning must anticipate the challenges that people might encounter in transitioning back to a satisfying life. Pandemic control measures can strain social relationships in various ways and people may need support in repairing those relationships. Clear guidance on how to socialize safely, and the importance of doing so, is also crucial to ensure members of the public can safeguard access to social support and thereby protect their physical and mental health—and the health of others.

## Supplementary Material

Supplemental_Material_formatted_refs_JPH_fdab394

## Data sharing

The data underlying the results reported in this article are not publicly available due to privacy or ethical restrictions. Deidentified data will be available following article publication upon reasonable request to the corresponding author. Requests should include Institutional Review Board approval for the reuse of the data.

## Notes

**Nicholas J. Long**, Associate Professor

**Nayantara Sheoran Appleton**, Senior Lecturer

**Sharyn Graham Davies**, Associate Professor

**Antje Deckert**, Associate Professor

**Edmond Fehoko**, Te Tomokanga Postdoctoral Fellow

**Eleanor Holroyd**, Professor of Nursing

**Nelly Martin-Anatias**, Research Fellow

**Rogena Sterling**, Research Officer

**Susanna Trnka**, Associate Professor

**Laumua Tunufa’i**, Lecturer
