# Supplementary material for: Pathways and obstacles to social recovery following the elimination of SARS-CoV-2 from Aotearoa New Zealand: a qualitative cross-sectional study
Source: J Public Health (Oxf). 2022 Jan 7;44(4):e548–56. doi: 10.1093/pubmed/fdab394 (PMC8807192; doi:10.1093/pubmed/fdab394)
Supplement: Supplemental_Material_formatted_refs_JPH_fdab394 [file supplemental_material_formatted_refs_jph_fdab394.docx]

Supplemental Material

**Table of Contents**

Annex 1: Schedule of survey questions 2

Annex 2: Descriptive statistical analysis of results 32

References 36

Annex 1: Schedule of Survey Questions

Start of Block: Default Question Block

Q1.1

**COVID-19 in 2021: Perspectives from New Zealand**

We are conducting a research project about what it’s like to live through the coronavirus pandemic, and would love to hear about your recent experiences of life in New Zealand.
  
This study involves answering a short survey. It usually takes no more than 20 minutes. 

The information you provide will be analysed by a small team of academic researchers based at the London School of Economics, Auckland University of Technology, The University of Auckland, The University of Waikato, and Victoria University of Wellington.
  
By taking part, you agree you have read and understood the following information about the study. 

**What is this project about, and do I have to take part?**

This survey is part of a larger study investigating the effects of the COVID-19 pandemic upon people living in New Zealand and elsewhere. The findings will be used to help develop ways to support people during this outbreak and future ones. Participation is open to people over the age of 18 living in New Zealand and is entirely voluntary.

**What will taking part involve?**

You will complete one online survey. You will be asked questions about yourself, your experiences of the pandemic so far, and your feelings about possible future scenarios. 
 At the end of the survey, we will ask you whether you might be willing to take part in follow-up research in future. If this is something you can help with, we will ask for an email address.

**Will my taking part and my data be kept confidential? Will it be anonymised?**

Unless you provide us with an email address for follow-up research, the data you provide will be wholly anonymous, and your participation will remain confidential even to us. If you do provide us with an email address, we will store this securely and delete it as soon as the project finished. We will not pass it to any third parties.  Nobody will be able to identify you from the anonymised data we analyse or from any publications based on this survey.

**How can I withdraw from the study?**

You may stop answering the survey at any time. If you have already submitted your answers, you may withdraw from the study at any time within fourteen calendar days  by contacting the Principal Investigator, Dr Nick Long ([N.J.Long@lse.ac.uk](mailto:N.J.Long@lse.ac.uk)). However, it will not be possible to remove your information from existing data sets once those data sets have been anonymised, or if you did not supply us with a contact email address, because even we would be unable to identify your data.

**Who has reviewed this study?**

This study has undergone ethics review in accordance with the LSE Research Ethics Policy and Procedure.

**Data Protection Privacy Notice**

The LSE Research Privacy Policy can be found at: [https://info.lse.ac.uk/staff/divisions/Secretarys- Division/Assets/Documents/Information-Records-Management/Privacy-Notice-for-Research-v1.1.pdf](https://info.lse.ac.uk/staff/divisions/Secretarys-%20Division/Assets/Documents/Information-Records-Management/Privacy-Notice-for-Research-v1.1.pdf) The legal basis used to process the personal data you provide will be Public Task. The legal basis used to process special category personal data will be for scientific and historical research or statistical purposes.

To request a copy of the data held about you please contact: [glpd.info.rights@lse.ac.uk](mailto:glpd.info.rights@lse.ac.uk)

**What if I have a question or complaint?**

If you have any questions regarding this study please contact the Principal Investigator, Dr Nick Long, on [N.J.Long@lse.ac.uk](mailto:N.J.Long@lse.ac.uk). If you have any concerns or complaints regarding the conduct of this research, please contact the LSE Research Governance Manager via [research.ethics@lse.ac.uk](mailto:research.ethics@lse.ac.uk)

Q1.2 **Consent Statement**
Please click on this statement to proceed

- I confirm that I am at least 18 years old, that I live in New Zealand, that I have read the information about the survey, and that I voluntarily agree to take part in this study. (1)

| Page Break |  |
| --- | --- |

Q1.3
 **We'd like to start with a few questions about you. These will allow us to see how representative our sample is. All questions are optional, so feel free to skip any you'd rather not answer.**

Q1.4 How old are you?

▼ 18 (1) ... 91 or older (74)

Q1.5 What is your gender?

- Man (1)
- Woman (2)
- Non-binary (3)
- Prefer not to say (4)

|  |
| --- |

Q1.6 Which of the following best describes your sexual orientation?

- Heterosexual (straight) (1)
- Homosexual (gay / lesbian) (2)
- Bisexual (3)
- Asexual (4)
- Other (please specify) (5) ________________________________________________
- Prefer not to say (6)

Q1.7 Which ethnicity / ethnicities do you identify with? Select all that apply.

- Māori (1)
- Pacific (2)
- European New Zealand/Pākeha (3)
- Asian (4)
- Middle Eastern (5)
- Latin American (6)
- African (7)
- Other (please specify) (8) ________________________________________________
- Prefer not to say (9)

Q1.8 Which of the following best describes your religious identity?

- Atheist (1)
- Agnostic (9)
- Christian (2)
- Muslim (3)
- Jewish (4)
- Buddhist (5)
- Hindu (6)
- Sikh (7)
- Not religious but spiritual (10)
- Other (please specify) (8) ________________________________________________
- Prefer not to say (11)

Q1.9 What is the highest level of education you have completed?

- No qualifications (1)
- Completed high school (2)
- Undergraduate degree or professional qualification (5)
- Postgraduate degree (6)
- Prefer not to say (7)

|  |
| --- |

Q1.10 What is your current employment status? Select all that apply.

- Employed (full-time) (1)
- Employed (part-time) (12)
- Self-employed (full-time) (2)
- Self-employed (part-time) (13)
- Unemployed (3)
- Looking after the family / home as a full-time job (5)
- Unpaid carer (6)
- In education (7)
- Long-term sick or disabled (8)
- Retired (9)
- Other (please specify) (10) ________________________________________________
- Prefer not to say (11)

| Page Break |  |
| --- | --- |

Q1.11 How many people are living in your household at present?

- 1 - it's just me! (1)
- 2 (2)
- 3 (3)
- 4 (4)
- 5 (5)
- 6 or more (6)

Q1.12 Are the people living in your household at the moment the people you usually live with?

- Yes (1)
- No, some people have joined because of the lockdown (2)
- No, some people are absent because of the lockdown (3)
- No, for reasons unrelated to the lockdown (4)
- Prefer not to say (5)

Q1.13 Does your bubble currently include anyone living in another household?

- Yes (please give details) (1) ________________________________________________
- No (2)
- Prefer not to say (3)

Display This Question:

If What is your current employment status? Select all that apply. = Employed (full-time)

Or What is your current employment status? Select all that apply. = Self-employed (full-time)

Or What is your current employment status? Select all that apply. = Employed (part-time)

Or What is your current employment status? Select all that apply. = Self-employed (part-time)

Q1.14 What is your job?

________________________________________________________________

Q1.15 It would help our research to know which part of the country you live in. If you feel comfortable doing so, please share your city, district, or postal code

________________________________________________________________

| Page Break |  |
| --- | --- |

Q1.16 Have you had COVID-19?

- Yes, confirmed by a test (1)
- No, not that I am aware (2)
- Not confirmed, but suspected (3)
- Prefer not to say (4)

Q1.17 Has anyone else in your current bubble had COVID-19?

- Yes, confirmed by a test (1)
- No, not that I am aware (2)
- Not confirmed, but suspected (3)
- Prefer not to say (4)

Q1.18 Do you have any underlying conditions that may affect your vulnerability to COVID-19?

- Yes (please give details if you feel comfortable doing so) (1) ________________________________________________
- No (2)
- Don't know (3)
- Prefer not to say (4)

Q1.19 Does anyone else in your current bubble have any underlying conditions that may affect their vulnerability to COVID-19?

- Yes (please give details if you feel comfortable doing so) (1) ________________________________________________
- No (2)
- Don't know (3)
- Prefer not to say (4)

Q1.20 Have you entered or left New Zealand at any time since 19th March 2020?

- Yes (1)
- No (2)
- Prefer not to say (3)

|  |
| --- |

Q1.21 Which of the following are currently your main sources of information about how New Zealand is handling COVID-19? Select all that apply.
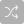


- National government website (1)
- Local council website (2)
- National news (TV, radio, papers, websites) (3)
- Local news (TV, radio, papers, websites) (4)
- Social media (5)
- Friends and family (6)
- Employer (7)
- Community leader (8)
- Other (please specify) (9) ________________________________________________
- I have not accessed any such information in recent months (10)

End of Block: Default Question Block

Start of Block: Attitudes

Q2.1
 **We'd now like to get an overview of your experiences of and attitudes towards different aspects of the pandemic.**

Q2.2
Please indicate how strongly you agree or disagree with each of the following statements:

Q2.3 Living in Level 4 lockdown was an unpleasant experience for me.

- Strongly agree (1)
- Somewhat agree (2)
- Neither agree nor disagree (3)
- Somewhat disagree (4)
- Strongly disagree (5)
- Not applicable / don't know / prefer not to say (6)

Q2.4 Catching COVID-19 would pose a serious risk to my health.

- Strongly agree (1)
- Somewhat agree (2)
- Neither agree nor disagree (3)
- Somewhat disagree (4)
- Strongly disagree (5)
- Don't know / prefer not to say (6)

Q2.5 Over the past six months, I have often worried that COVID-19 is circulating in my community undetected.

- Strongly agree (1)
- Somewhat agree (2)
- Neither agree nor disagree (3)
- Somewhat disagree (4)
- Strongly disagree (5)
- Don't know / prefer not to say (6)

Q2.6 I feel confident that vaccinations against COVID-19 are safe.

- Strongly agree (1)
- Somewhat agree (2)
- Neither agree nor disagree (3)
- Somewhat disagree (4)
- Strongly disagree (5)
- Don't know / prefer not to say (6)

| Page Break |  |
| --- | --- |

Q2.7 My household finances have been badly affected by the COVID-19 pandemic.

- Strongly agree (1)
- Somewhat agree (2)
- Neither agree nor disagree (3)
- Somewhat disagree (4)
- Strongly disagree (5)
- Don't know / prefer not to say (6)

Q2.8 My life has been badly affected by New Zealand's border restrictions.

- Strongly agree (1)
- Somewhat agree (2)
- Neither agree nor disagree (3)
- Somewhat disagree (4)
- Strongly disagree (5)
- Don't know / prefer not to say (6)

Q2.9 Overall, New Zealand has handled COVID-19 better than most other countries.

- Strongly agree (1)
- Somewhat agree (2)
- Neither agree nor disagree (3)
- Somewhat disagree (4)
- Strongly disagree (5)
- Don't know / prefer not to say (6)

End of Block: Attitudes

Start of Block: Snap lockdown

Q3.1
  **The whole of New Zealand entered Level 4 lockdown at 23:59 on 17th August 2021.**

Q3.2
Do you support or oppose the re-introduction of a Level 4 lockdown?

- Strongly support (1)
- Somewhat support (2)
- Neither support nor oppose (3)
- Somewhat oppose (4)
- Strongly oppose (5)
- Prefer not to say (6)

Q3.3 How well prepared were you for this Level 4 lockdown?

- Extremely well prepared (1)
- Somewhat well prepared (2)
- Neither well prepared nor unprepared (3)
- Somewhat unprepared (4)
- Extremely unprepared (5)
- Prefer not to say (6)

Q3.4
Before this lockdown was announced, how much had you discussed who would be in your bubble if New Zealand returned to Level 4?

- A great deal (1)
- A little (2)
- Not very much (3)
- Not at all (4)
- Prefer not to say (6)

Q3.5 Please explain your answers

________________________________________________________________

________________________________________________________________

________________________________________________________________

________________________________________________________________

________________________________________________________________

| Page Break |  |
| --- | --- |

Q3.6
  **Imagine that this lockdown ended up lasting several months...**

Q3.7 What, if anything, would you do differently during this lockdown compared to the last Level 4 lockdown?

________________________________________________________________

________________________________________________________________

________________________________________________________________

________________________________________________________________

________________________________________________________________

Q3.8 Are there any additional forms of support you would hope to receive from the government, compared to the last Level 4 lockdown?

________________________________________________________________

________________________________________________________________

________________________________________________________________

________________________________________________________________

________________________________________________________________

End of Block: Snap lockdown

Start of Block: Travel and MIQ

Display This Block:

If Have you entered or left New Zealand at any time since 19th March 2020? = Yes

Q4.1
 **Earlier, you indicated that you had travelled into or out of New Zealand during the pandemic.**
 
Where did you travel to / from, and for what reasons?

________________________________________________________________

________________________________________________________________

________________________________________________________________

________________________________________________________________

________________________________________________________________

Q4.2 Did any of your travel require you to stay in Managed Isolation and Quarantine (MIQ) facilities?

- Yes (1)
- No (2)
- Prefer not to say (3)

Q4.3 How did you find the experience of travelling during the pandemic? 
We are interested in hearing about positive and negative aspects of your travel experience (including your stay in MIQ facilities, where applicable).

________________________________________________________________

________________________________________________________________

________________________________________________________________

________________________________________________________________

________________________________________________________________

Q4.4 What changes could be made to better support people wishing to travel out of, or into, New Zealand during the pandemic?

________________________________________________________________

________________________________________________________________

________________________________________________________________

________________________________________________________________

________________________________________________________________

End of Block: Travel and MIQ

Start of Block: Borders

Q5.1
  **We would now like to ask some more about border controls.**

Display This Question:

If My life has been badly affected by New Zealand's border restrictions. = Strongly agree

Or My life has been badly affected by New Zealand's border restrictions. = Somewhat agree

Q5.2 Earlier, you indicated that you had been badly affected by New Zealand's border restrictions. Could you tell us more about how the border restrictions have affected you?

________________________________________________________________

________________________________________________________________

________________________________________________________________

________________________________________________________________

________________________________________________________________

Q5.3 How, if at all, have other people that you know been affected by New Zealand's border restrictions?

________________________________________________________________

________________________________________________________________

________________________________________________________________

________________________________________________________________

________________________________________________________________

Q5.4 What more could the New Zealand government do to support people whose lives are being badly affected by New Zealand's border restrictions?

________________________________________________________________

________________________________________________________________

________________________________________________________________

________________________________________________________________

________________________________________________________________

| Page Break |  |
| --- | --- |

Q5.5 On a scale of 0 to 5, how detailed is your knowledge of New Zealand's current strategy for reopening its borders?

- 0 - I don't know anything about this (1)
- 1 - I know very little about this (2)
- 2 - I know a little about this (3)
- 3 - I know a reasonable amount about this (4)
- 4 - I know a lot about this (5)
- 5 - I know a very great deal about this (6)
- Prefer not to say (7)

| Page Break |  |
| --- | --- |

Display This Question:

If On a scale of 0 to 5, how detailed is your knowledge of New Zealand's current strategy for reopen... = 3 - I know a reasonable amount about this

Or On a scale of 0 to 5, how detailed is your knowledge of New Zealand's current strategy for reopen... = 4 - I know a lot about this

Or On a scale of 0 to 5, how detailed is your knowledge of New Zealand's current strategy for reopen... = 5 - I know a very great deal about this

Q5.6 How satisfied are you with the government's current plans for reopening New Zealand's borders?

- Very satisfied (1)
- Somewhat satisfied (2)
- Neither satisfied nor dissatisfied (3)
- Somewhat dissatisfied (4)
- Very dissatisfied (5)
- Don't know / prefer not to say (6)

Display This Question:

If On a scale of 0 to 5, how detailed is your knowledge of New Zealand's current strategy for reopen... = 3 - I know a reasonable amount about this

Or On a scale of 0 to 5, how detailed is your knowledge of New Zealand's current strategy for reopen... = 4 - I know a lot about this

Or On a scale of 0 to 5, how detailed is your knowledge of New Zealand's current strategy for reopen... = 5 - I know a very great deal about this

Q5.7 What, if anything, do you think should be done differently?

________________________________________________________________

________________________________________________________________

________________________________________________________________

________________________________________________________________

| Page Break |  |
| --- | --- |

Q5.8 **To what extent do you agree with the following statements:**

Q5.9 New Zealand's borders should remain closed until at least the end of 2021.

- Strongly agree (1)
- Somewhat agree (2)
- Neither agree nor disagree (3)
- Somewhat disagree (4)
- Strongly disagree (5)
- Prefer not to say (6)

Q5.10 New Zealand's borders should remain closed until COVID-19 is under control everywhere in the world.

- Strongly agree (1)
- Somewhat agree (2)
- Neither agree nor disagree (3)
- Somewhat disagree (4)
- Strongly disagree (5)
- Prefer not to say (6)

Q5.11 Athletes should be allowed to travel in and out of New Zealand to take part in international sporting events.

- Strongly agree (1)
- Somewhat agree (2)
- Neither agree nor disagree (3)
- Somewhat disagree (4)
- Strongly disagree (5)
- Prefer not to say (6)

Q5.12 The capacity of New Zealand's Managed Isolation and Quarantine (MIQ) facilities should be expanded.

- Strongly agree (1)
- Somewhat agree (2)
- Neither agree nor disagree (3)
- Somewhat disagree (4)
- Strongly disagree (5)
- Prefer not to say (6)

Q5.13 When the borders re-open, only fully vaccinated people should be allowed to enter New Zealand.

- Strongly agree (1)
- Somewhat agree (2)
- Neither agree nor disagree (3)
- Somewhat disagree (4)
- Strongly disagree (5)
- Prefer not to say (6)

Q5.14 If you would like to, please explain your answers

________________________________________________________________

________________________________________________________________

________________________________________________________________

________________________________________________________________

________________________________________________________________

End of Block: Borders

Start of Block: Before and after

Q6.1
  **We would now like to ask about some other ways in which your life may have changed since the start of the COVID-19 pandemic.**

Q6.2 Over the past six months, have your **relationships with people who were in your lockdown bubble** been similar or different to how they were before the pandemic?

- More or less the same (1)
- A little different (2)
- Extremely different (3)

Q6.3 Please explain your answer

________________________________________________________________

________________________________________________________________

________________________________________________________________

________________________________________________________________

________________________________________________________________

Q6.4 Over the past six months, have your **friendships and social life** been similar or different to how they were before the pandemic?

- More or less the same (1)
- A little different (2)
- Extremely different (3)

Q6.5 Please explain your answer.

________________________________________________________________

________________________________________________________________

________________________________________________________________

________________________________________________________________

________________________________________________________________

| Page Break |  |
| --- | --- |

Q6.6 Over the past six months, has the current **atmosphere in your local community / neighbourhood**been similar or different to how it was before the pandemic?

- More or less the same (1)
- A little different (2)
- Extremely different (3)

Q6.7 Please explain your answer.

________________________________________________________________

________________________________________________________________

________________________________________________________________

________________________________________________________________

________________________________________________________________

Display This Question:

If What is your current employment status? Select all that apply. = Employed (full-time)

Or What is your current employment status? Select all that apply. = Self-employed (full-time)

Or What is your current employment status? Select all that apply. = Employed (part-time)

Or What is your current employment status? Select all that apply. = Self-employed (part-time)

Q6.8 Over the past six months, has your **working life**been similar or different to how it was before the pandemic?

- More or less the same (1)
- A little different (2)
- Extremely different (3)

Display This Question:

If What is your current employment status? Select all that apply. = Employed (full-time)

Or What is your current employment status? Select all that apply. = Employed (part-time)

Or What is your current employment status? Select all that apply. = Self-employed (full-time)

Or What is your current employment status? Select all that apply. = Self-employed (part-time)

Q6.9 Please explain your answer.

________________________________________________________________

________________________________________________________________

________________________________________________________________

________________________________________________________________

________________________________________________________________

Q6.10 Over the past six months, has your**outlook on life**been similar or different to how it was before the pandemic?

- More or less the same (1)
- A little different (2)
- Extremely different (3)

Q6.11 Please explain your answer.

________________________________________________________________

________________________________________________________________

________________________________________________________________

________________________________________________________________

________________________________________________________________

End of Block: Before and after

Start of Block: Vaccination

Q7.1
 **We would now like to ask you some questions regarding vaccinations against COVID-19.**

Q7.2 What are your personal plans regarding vaccination?

- I have already had at least one dose of vaccine (1)
- I plan to get vaccinated as soon as I am able (2)
- I am not sure whether to get vaccinated (3)
- I am not planning to get vaccinated (4)
- Prefer not to say (5)

Q7.3 In your opinion, how important is it that people in New Zealand be offered a choice as to which COVID-19 vaccine they receive?

- Extremely important (1)
- Very important (2)
- Moderately important (3)
- Not very important (4)
- Not at all important (5)
- Prefer not to say (6)

Q7.4 In your opinion, how important is it that New Zealand secures enough supplies of vaccines for every citizen to be offered multiple booster doses during 2022?

- Extremely important (1)
- Very important (2)
- Moderately important (3)
- Not very important (4)
- Not at all important (5)
- Prefer not to say (6)

Q7.5 If you would like to, please explain your answers

________________________________________________________________

________________________________________________________________

________________________________________________________________

________________________________________________________________

________________________________________________________________

| Page Break |  |
| --- | --- |

Q7.6 Do you agree with the following statements:

Q7.7 Vaccinations against COVID-19 should be made compulsory in New Zealand.

- Yes, for everyone who is eligible (unless they are medically exempt) (1)
- Only for certain groups of people (2)
- No (3)
- Don't know (4)
- Prefer not to say (5)

Q7.8 Employers in New Zealand should be allowed to insist that their employees be vaccinated against COVID-19.

- Yes (1)
- Only for certain sectors (2)
- No (3)
- Don't know (4)
- Prefer not to say (5)

Q7.9 Businesses in New Zealand should be allowed to turn away unvaccinated customers and clients.

- Yes (1)
- Only for certain sectors (2)
- No (3)
- Don't know (4)
- Prefer not to say (5)

Q7.10 If you would like to, please explain your answers.

________________________________________________________________

________________________________________________________________

________________________________________________________________

________________________________________________________________

________________________________________________________________

End of Block: Vaccination

Start of Block: Future scenarios

Q8.1
 **Finally, we would like to ask you about a possible future scenario.**
 
 
First, imagine that everyone in New Zealand who wishes to be fully vaccinated already has been. COVID-19 cases are then detected in the community.

Q8.2 What changes (if any) would you make to your everyday life in such a scenario, and why?

________________________________________________________________

________________________________________________________________

________________________________________________________________

________________________________________________________________

________________________________________________________________

Q8.3 What action should the government take in such a scenario, and why?

________________________________________________________________

________________________________________________________________

________________________________________________________________

________________________________________________________________

________________________________________________________________

End of Block: Future scenarios

Start of Block: Other countries

Q9.1 Earlier, you indicated that you did not feel New Zealand had the pandemic better than most other countries.


Which countries have handled the pandemic better than (or as well as) New Zealand, and why?

________________________________________________________________

________________________________________________________________

________________________________________________________________

________________________________________________________________

________________________________________________________________

End of Block: Other countries

Start of Block: End

Q10.1 You're almost at the end of the survey. Before you finish, are there any other insights you would like to share with us about your experiences of living through the pandemic in 2021 - or your hopes and fears for the future?

________________________________________________________________

________________________________________________________________

________________________________________________________________

________________________________________________________________

________________________________________________________________

Display This Question:

If Contact List ExternalDataReference Is Empty

Q10.2 Thank you so much for taking the time to fill in this survey. Your answers will really help us understand how people in New Zealand are experiencing this current phase of the pandemic.


Would you be happy for us to contact you about future opportunities to take part in research on how your life has been affected by COVID-19?

- Yes (1)
- No (2)

Display This Question:

If Thank you so much for taking the time to fill in this survey. Your answers will really help us un... = Yes

Q10.3 Fantastic - thank you!


Please let us know the best email address for us to contact you

________________________________________________________________

End of Block: End

**Annex 2: Descriptive Statistical Analysis of Results**

The coding strategy employed in our thematic analysis allowed us to conduct a statistical exploration of patterns in the data.^1^ Since the survey sample was not selected randomly, this analysis was descriptive and exploratory of our respondent pool rather than intended to make inferential claims about the parent population (i.e. all people living in New Zealand).^2^ Following the example of other medical and social science researchers who have used statistical tests to explore coded qualitative data,^3-5^ we conducted Chi-squared tests for independence to examine the frequency of three key themes – ‘returning to normal’ (code N), ‘becoming more social’ (code P), and ‘becoming less social’ (code L) – in relation to gender, ethnicity, age, education status, household size, and presence or absence of underlying health conditions that might affect one’s vulnerability to COVID-19. In cases where responses had received multiple codes, we identified a single ‘predominant’ code for the purposes of statistical analysis, since the Chi-square test requires independent observations. Recognising that respondents may have been less likely to elaborate on an answer indicating that their lives were ‘more or less the same’ than when having reported a difference, separate tests were run for associations between the prevalence of P and L codes not only vis-à-vis N codes but also vis-à-vis N codes *and* unelaborated ‘more or less the same’ answers. Responses in which the participant had not provided demographic information or indicated that they ‘preferred not to answer’ were excluded from the statistical analysis (although still included in the thematic analysis).

**Distribution of response codings in relation to gender**

| **Gender** | **'No Change' codings** | **'No Change' codings *and* unelaborated 'More or Less the Same' answers** | **'Less Social' codings** | **'More Social' codings** |
| --- | --- | --- | --- | --- |
| Woman | 100 | 400 | 203 | 78 |
| Man | 26 | 101 | 32 | 7 |
| Non-binary | 2 | 7 | 8 | 1 |
| Prefer not to say / blank | 1 | 5 | 4 | 0 |

When the responses of non-binary respondents were included in the analysis, there was no statistically significant association between gender and the respective prevalence of ‘No Change’, ‘Less Social’ and ‘More Social’ codings (Χ*²* [4, N=457] = 8·465; p=0·076), although there was a statistically significant association with gender when the prevalence of ‘Less Social’ and ‘More Social’ codings was compared with that of ‘No Change’ codings *and* unelaborated ‘More or less the same’ answers (Χ² [4, N=837] = 13·578; p=0·0088). When non-binary responses were excluded (due to small cell sizes), statistically significant association were found between gender and the respective prevalence of ‘No Change’, ‘Less Social’ and ‘More Social’ codings (Χ² [2, N=446] = 6·534; p=0·036), as well as between gender and the respective prevalence of Less Social’ and ‘More Social’ codings vis-à-vis No Change’ codings *and* unelaborated ‘More or less the same’ answers (Χ² [2, N=821] = 10·052; p=0·0066).

**Distribution of response codings in relation to ethnicity**

| **Ethnicity** | **'No Change' codings** | **'No Change' codings *and* unelaborated 'More or Less the Same' answers** | **'Less Social' codings** | **'More Social' codings** |
| --- | --- | --- | --- | --- |
| New Zealand European / Pākehā | 104 | 459 | 215 | 85 |
| White Other | 5 | 25 | 20 | 6 |
| Māori | 12 | 34 | 22 | 3 |
| Pacific | 6 | 12 | 5 | 2 |
| Asian | 5 | 18 | 5 | 3 |
| Other | 1 | 8 | 1 | 1 |
| Prefer not to say / blank | 5 | 11 | 8 | 0 |

Research participants were able to indicate multiple identifications in the survey, as is also the case in the New Zealand census, and as reflects the complex and dynamic nature of ethnic identification and cultural affiliation in contemporary New Zealand.^6,7^ Since Chi-squared tests require independent observations, we concatenated the data for statistical analysis. We ran separate tests to see whether there were any associations between survey responses and identifying exclusively as White (whether by checking the ‘New Zealand European / Pākehā’ box, or reporting another Caucasian ethnicity under ‘Other’) or identifying non-exclusively as Māori. We also tested to see whether there was any association with being Māori *or* Pacific, since these two groups have been identified as especially vulnerable to COVID-19 and affected by longstanding structural inequalities.^8-11^ Small cell sizes precluded tests for associations with other ethnicity options in the survey.

**Distribution of response codings in relation to concatenated ethnicity**

| **Concatenated ethnicity** | **'No Change' Codings** | **No Change' codings *and* unelaborated 'More or Less the Same' answers** | **'Less Social' Codings** | **'More Social' Codings** |
| --- | --- | --- | --- | --- |
| White only | 99 | 452 | 212 | 84 |
| Person of Colour | 22 | 68 | 33 | 11 |
| Māori | 12 | 34 | 22 | 3 |
| Non-Māori | 109 | 486 | 223 | 92 |
| Māori and Pacific | 17 | 43 | 27 | 5 |
| Neither Māori nor Pacific | 104 | 477 | 218 | 90 |

There was no statistically significant association between the respective prevalence of ‘No Change’, ‘Less Social’ and ‘More Social’ codings and being White versus being a person of colour (Χ² [2, N=461] = 2·197; p=0·33), being Māori versus being non-Māori (Χ² [2, N=461] = 3·938; p=0·14) or being either Māori or Pacific versus being neither (Χ² [2, N=461] = 4·409; p=0·11). When the tests were broadened to include unelaborated ‘more of less the same’ answers as well as ‘No Change’ codings, there were still no statistically significant associations with being White versus being a person of colour (Χ² [2, N=860] = 0·219; p=0·90), being Māori versus being non-Māori (Χ² [2, N=860] = 3·844; p=0·15), or being either Māori or Pacific versus being neither (Χ² [2, N=860] = 3·188; p=0·20).

**Distribution of response codings in relation to age**

| **Age** | **'No Change' codings** | **'No Change' codings *and* unelaborated 'More or Less the Same' answers** | **'Less Social' codings** | **'More Social' codings** |
| --- | --- | --- | --- | --- |
| 18-34 | 31 | 93 | 38 | 24 |
| 35-50 | 32 | 142 | 66 | 23 |
| 50-64 | 43 | 193 | 80 | 24 |
| 65+ | 23 | 77 | 59 | 11 |
| Prefer not to say / blank | 0 | 8 | 4 | 4 |

There was no statistically significant association between the respective prevalence of ‘No Change’, ‘Less Social’ and ‘More Social’ codings and age (Χ² [6, N=454] = 11·372; p=0·078). However, when the test was broadened to include unelaborated ‘more of less the same’ answers as well as ‘No Change’ codings, a statistically significant association with age could be observed (Χ² [6, N=830] = 17·072; p=0·0090).

**Distribution of response codings in relation to education status**

| **Education status** | **'No Change' codings** | **'No Change' codings *and* unelaborated 'More or Less the Same' answers** | **'Less Social' codings** | **'More Social' codings** |
| --- | --- | --- | --- | --- |
| No qualifications / high school | 22 | 90 | 29 | 12 |
| Undergraduate degree / professional qualification | 70 | 248 | 111 | 43 |
| Postgraduate degree | 37 | 172 | 99 | 31 |
| Prefer not to say / blank | 0 | 3 | 2 | 0 |

There was no statistically significant association between the respective prevalence of ‘No Change’, ‘Less Social’ and ‘More Social’ codings and education status (Χ² [4, N=460] = 6·223; p=0·18). When the test was broadened to include unelaborated ‘more of less the same’ answers as well as ‘No Change’ codings, there was still no statistically significant association with education status (Χ² [4, N=841] = 6·233; p=0·18).

**Distribution of response codings in relation to household size**

| **Household size** | **'No Change' codings** | **'No Change' codings *and* unelaborated 'More or Less the Same' answers** | **'Less Social' codings** | **'More Social' codings** |
| --- | --- | --- | --- | --- |
| 1 | 27 | 102 | 49 | 19 |
| 2 | 41 | 173 | 88 | 27 |
| 3 | 23 | 102 | 44 | 18 |
| 4 | 23 | 91 | 34 | 13 |
| 5 or more | 15 | 45 | 31 | 9 |
| Prefer not to say / blank | 0 | 0 | 1 | 0 |

There was no statistically significant association between the respective prevalence of ‘No Change’, ‘Less Social’ and ‘More Social’ codings and household size (Χ² [8, N=461] = 2·249; p=0·97). When the test was broadened to include unelaborated ‘more of less the same’ answers as well as ‘No Change’ codings, there was still no statistically significant association with household size (Χ² [8, N=845] = 5·138; p=0·74).

**Distribution of response codings in relation to self-reported health status (presence or absence of an underlying condition that would heighten vulnerability to COVID-19)**

| **Underlying health condition** | **'No Change' codings** | **'No Change' codings *and* unelaborated 'More or Less the Same' answers** | **'Less Social' codings** | **'More Social' codings** |
| --- | --- | --- | --- | --- |
| Yes | 31 | 144 | 107 | 31 |
| No | 91 | 340 | 122 | 52 |
| Don't know | 6 | 25 | 14 | 3 |
| Prefer not to say / blank | 1 | 4 | 4 | 0 |

Even when ‘don’t know’ answers were included in the analysis, there was a statistically significant association between the respective prevalence of ‘No Change’, ‘Less Social’ and ‘More Social’ codings and self-reported health status (Χ² [4, N=457] = 16·040; p=0·0030). When the test was broadened to include unelaborated ‘more of less the same’ answers as well as ‘No Change’ codings, this association was even more clearly in evidence (Χ² [4, N=838] = 20·350; p=0·00043). When ‘don’t know’ answers were excluded, p values became smaller still, both when testing for associations between the respective prevalence of ‘No Change’, ‘Less Social’ and ‘More Social’ codings and self-reported health status (Χ² [2, N=434] = 15·318; p=0·00047), and when broadening the test to include unelaborated ‘more of less the same’ answers as well as ‘No Change’ codings (Χ² [2, N=796] = 19·725; p<0·0001).

**References**

1. Joffe H, Yardley L. Content and thematic analysis. In: Marks DF, Yardley L, eds. Research Methods for Clinical and Health Psychology. London: SAGE; 2004: 56-69.

2. Guest G, MacQueen KM, Namey EE. Applied Thematic Analysis. Los Angeles: SAGE; 2012.

3. Galupo MP, Ramirez JL, Pulice-Farrow L. “Regardless of Their Gender”: Descriptions of Sexual Identity among Bisexual, Pansexual, and Queer Identified Individuals. *J Bisex* 2017; **17**(1): 108-24.

4. Hochard KD, Ashcroft S, Carroll J, Heym N, Townsend E. Exploring Thematic Nightmare Content and Associated Self-Harm Risk. *Suicide Life Threat Behav* 2019; **49**(1): 64-75.

5. Bernard AW, Malone M, Kman NE, Caterino JM, Khandelwal S. Medical Student Professionalism Narratives: A Thematic Analysis and Interdisciplinary Comparative Investigation. *BMC Emerg Med* 2011; **11**: 11.

6. Boven N, Exeter D, Sporle A, Shackleton N. The implications of different ethnicity categorisation methods for understanding outcomes and developing policy in New Zealand. *Kōtuitui: N Z J Soc Sci* 2020; **15**(1): 123-39.

7. Cormack D, Robson C. Classification and output of multiple ethnicities: issues for monitoring Māori health. Wellington: Te Rōpū Rangahau Hauora a Eru Pōmare; 2010.

8. Steyn N, Binny RN, Hannah K, et al. Māori and Pacific people in New Zealand have a higher risk of hospitalisation for COVID-19. *N Z Med J* 2021; **134**(1538): 28-43.

9. Marriott L, Alinaghi N. Closing the Gaps: An Update on Indicators of Inequality for Māori and Pacific People. *J N Z Stud* 2021; **NS32**: 2-39.

10. Marriott L, Sim D. Indicators of Inequality for Māori and Pacific People. Wellington: Victoria University of Wellington Working Papers in Public Finance; 2014.

11. Moewaka Barnes H, McCreanor T. Colonisation, hauora and whenua in Aotearoa. *J R Soc N Z* 2019; **49**(sup1): 19-33.
